# Supplementary material for: Rapid turnover of life-cycle-related genes in the brown algae
Source: Genome Biol. 2019 Feb 14;20:35. doi: 10.1186/s13059-019-1630-6 (PMC6374913; doi:10.1186/s13059-019-1630-6)
Supplement: Supplementary file 3 — Protein alignments, corresponding Gblocks html file and CDS sequences for the lineage conserved gametophyte-biased genes. (ZIP 2109 kb) [file 13059_2019_1630_MOESM3_ESM.zip › Con_GA_alignments/Ec-01_007620.aa_ali.fasta-gb.htm]

Ec-01\_007620.aa.fasta.trans.aa\_ali.fasta


## Gblocks 0.91b Results

Processed file: **Ec-01\_007620.aa.fasta.trans.aa\_ali.fasta**  
Number of sequences: **2**  
Alignment assumed to be: **Protein**  
New number of positions: **676** (selected positions are underlined in blue)

```
                         10        20        30        40        50        60
                 =========+=========+=========+=========+=========+=========+
Ec-01_007620     MTHVQRRCTFTHLIFLGCLGCLGSPTSSFTVESRGVLRNVALHAQRPPSPQAVVGSAVTM
MSTRG.47936.1    MAPDIRIQSFGCHAFLRLFGCF-----SFCRCAGGGGINVALHAQARASKEMVVGSAVTM
                 #####################      #################################


                         70        80        90       100       110       120
                 =========+=========+=========+=========+=========+=========+
Ec-01_007620     AGVRQALLRRDWKISDNATSSGRKDSGGSTVEGGRSSGEGTKITQQEDQPHVRIFYPFNY
MSTRG.47936.1    AGVRQALLTRDERKNGGAFMRDK-----------EDGGGGFADVK---TENVTVFYPFDY
                 ##################                                 #########


                        130       140       150       160       170       180
                 =========+=========+=========+=========+=========+=========+
Ec-01_007620     EGLHARSWDLVIIEGWFGMINSFIHEVRAITHPHSVTVLFYCLDPEYPGLPHVAALDVEG
MSTRG.47936.1    GGLHDRSWDLVIIEGWFGMINAFIHEVRAISHPRPVTILFYCLDPQYPGLSRVAALDVEG
                 ############################################################


                        190       200       210       220       230       240
                 =========+=========+=========+=========+=========+=========+
Ec-01_007620     FLTNSESTRQYLRQSAPTALVPLAVDTEAFPFYPDPPRPRAAAAAITSSRGHPYEDNGGG
MSTRG.47936.1    FLTNSESTRRFLVSAAPTAYVPLAADTEAFPFHPIPPCP-SGAVEGTATRRCPSEDDCRV
                 #######################################   ##################


                        250       260       270       280       290       300
                 =========+=========+=========+=========+=========+=========+
Ec-01_007620     DDAFVGRWDRTSHGRVVYVGSATLMEHKEMLPWMLREAAPFGLDIYGNGWQEFPEFSEYW
MSTRG.47936.1    EDVFGGGGNASRYGRVVYVGAAELIEYKEMLPRMLREAAPYGLDIYGSGWRNYPEFSGYW
                 ############################################################


                        310       320       330       340       350       360
                 =========+=========+=========+=========+=========+=========+
Ec-01_007620     RGVLPQEDLAFVYGSALAVVGVTTDAQRGSGKINNRVYEALSVGAPLISDHFPALEATFG
MSTRG.47936.1    RGVLPQEDLAFVYGTALAVIGVTTDAQRESGKINNRVYEVLSVGAPLVSDYFLALEAIFG
                 ############################################################


                        370       380       390       400       410       420
                 =========+=========+=========+=========+=========+=========+
Ec-01_007620     DALLYARRRGDVARHIESLLQSQQAVGCDNTEASNRRQRRAMIEAGHTWSQRVEDILSFV
MSTRG.47936.1    DSIFYARSPGDVARHIETILHSCRAPGQNRAEADERRRRRAIIENSHTWSQRSEDILAFA
                 ############################################################


                        430       440       450       460       470       480
                 =========+=========+=========+=========+=========+=========+
Ec-01_007620     GSLHGNSAATAFVEDEGEGGRG---EEAARCSRQRGCLTVAIVVDHELEGDITFESTFVP
MSTRG.47936.1    ESLPGPLAIARVPDSDASGKRGRSGPSVARCTRQGGCLSIAIVVDHDLDGDITFLSTFVP
                 ########                    ################################


                        490       500       510       520       530       540
                 =========+=========+=========+=========+=========+=========+
Ec-01_007620     AVDLLLGSTYLFSWFTSPQPWGGARGPNRTTADRMLEREQEEENKHKANGASYRKRRIMR
MSTRG.47936.1    AVD-LLGSAYRITWWMTPTSWDGDYGDNRTTTR----PQEEEPARHRAGGATFGKRRHMQ
                 ### ###########################         ####################


                        550       560       570       580       590       600
                 =========+=========+=========+=========+=========+=========+
Ec-01_007620     LPRDADCLSEYDVVWATGRWGGTADRTVRELLQPAGTAATRRGSTSRLMAQLKGMVLWGP
MSTRG.47936.1    LPRDSGCLEKYDAVWAAGQWGGTADRIVRTLSRRMGTVSTRRATTPRLTAQLTGLVLWGF
                 ############################################################


                        610       620       630       640       650       660
                 =========+=========+=========+=========+=========+=========+
Ec-01_007620     LCTPRTGSEADGEGREPEPDVRDYGKGCPGYAGREGLRWYDVVYCQTNGEHDFLLQQAFE
MSTRG.47936.1    LCTPSTNSPGDQEGDRPGSGASDREESCPDFAGQAGLRWYDVVYCQTGWDHAFLTRQAFE
                 ############################################################


                        670       680       690       700       710       720
                 =========+=========+=========+=========+=========+=========+
Ec-01_007620     GDVSDNLQQAWGYGIAHGSRSNGVPVDGDGDQGTAAVSTHPLDMLVVGTDDQVPDMLRVL
MSTRG.47936.1    GEVSSNLQQAWGFGTALKTRSDGIYRSGDE---MAVSSTQQLDVLVVGTDSQLPDMLGYL
                 #############################     ##########################


                        730       740       750       760       770       780
                 =========+=========+=========+=========+=========+=========+
Ec-01_007620     NTPGLGRVALSVVVTPGGADATAAARPGLASILAAAGVDSKMRIDDLPQRLSVYVVDSSE
MSTRG.47936.1    KVPGVRHIALAVIASTTGA---IEANSVLRRILGAAGVX---------------------
                 ###################     ##############                      


                        790       800       810       820       830       840
                 =========+=========+=========+=========+=========+=========+
Ec-01_007620     GSSFTPLITEVFLVHRAADADALAEISSRAAKVAVLAVGQLGAWAVVVSTSEVHEARWRR
MSTRG.47936.1    -PWCT-------------------------------------------------------
                                                                             


                        850       860       870       880       890       900
                 =========+=========+=========+=========+=========+=========+
Ec-01_007620     RFDVREFDIGDSDRLRALIQERPAAWDADYYSRRLVAGMTRALCLGRGNSRISFVRPASE
MSTRG.47936.1    ------------------------------------------------------------
                                                                             


                        910       920       930       940       950       960
                 =========+=========+=========+=========+=========+=========+
Ec-01_007620     SPAEVVGAINTSITVEVLVGDFHVGRDGEWCITVQGRTALCVLQNVFVVDIHVSSSASKS
MSTRG.47936.1    ------------------------------------------------------------
                                                                             


                        970       980       990      1000      1010      1020
                 =========+=========+=========+=========+=========+=========+
Ec-01_007620     DWEGVLATAGLKSGKQDDSKRENGVETCTKGTICARQLQQGFVRVEVRAELRSNMYKDVL
MSTRG.47936.1    ------------------------------------------------------------
                                                                             


                       1030      1040      1050      1060      1070      1080
                 =========+=========+=========+=========+=========+=========+
Ec-01_007620     QRSKPFYMYIDPIGAELADVRCADDRNASFVGDEVGRGAFQTSIDVWEYLESGSIVALEV
MSTRG.47936.1    ------------------------------------------------------------
                                                                             


                       1090
                 =========+========
Ec-01_007620     ARPYGKDTLSHREEVITT
MSTRG.47936.1    -----------------P
```

```
Parameters used
Minimum Number Of Sequences For A Conserved Position: 2
Minimum Number Of Sequences For A Flanking Position: 2
Maximum Number Of Contiguous Nonconserved Positions: 8
Minimum Length Of A Block: 10
Allowed Gap Positions: None
Use Similarity Matrices: Yes
```

```
Flank positions of the 9 selected block(s)
Flanks: [1  21]  [28  78]  [112  219]  [223  428]  [449  483]  [485  511]  [521  689]  [695  739]  [745  758]  

New number of positions in Ec-01_007620.aa.fasta.trans.aa_ali.fasta-gb:  676  (61% of the original 1098 positions)
```
